# Supplementary material for: Mucosal Melanoma Clinical Management and Prognostic Implications: A Retrospective Cohort Study
Source: Cancers (Basel). 2024 Jan 3;16(1):227. doi: 10.3390/cancers16010227 (PMC10778057; doi:10.3390/cancers16010227)

cancers

MDPI

Article

Mucosal Melanoma Clinical Management and Prognostic Implications: A Retrospective Cohort Study

Laia Clavero-Rovira[1], Álvaro Gómez-Tomás[2], Patricia Bassas[3], Domingo Bodet[4], Berta Ferrer[5], Javier Hernández-Losa[6], Eva Muñoz-Couselo[7], Maria Asunción Pérez-Benavente[8], Vicente García-Patos[9], Carla Ferrándiz-Pulido[10,*]

Supplementary Materials:

Table S1: Clinical and tumor characteristic by disease stage.

	Localized (N=11)	Nodal (N=13)	Distant (N=11)	Total (N=35)	P-value
**Sex**					0.486
Female	6 (54.5%)	10 (76.9%)	6 (54.5%)	22 (62.9%)	
Male	5 (45.5%)	3 (23.1%)	5 (45.5%)	13 (37.1%)	
**Age at diagnosis, years**					0.001
Median (Q1,Q3)	83.0 (78.5, 85.5)	67.0 (57.0, 82.0)	63.0 (55.5, 67.5)	70.0 (60.5, 82.0)	
**Smoking, ever**					0.021
No	6 (66.7%)	8 (61.5%)	1 (10.0%)	15 (46.9%)	
Yes	3 (33.3%)	5 (38.5%)	9 (90.0%)	17 (53.1%)	
Unknown	2	0	1	3	
**Immunosuppression**					1.000
No	11 (100.0%)	12 (92.3%)	10 (90.9%)	33 (94.3%)	
Yes	0 (0.0%)	1 (7.7%)	1 (9.1%)	2 (5.7%)	
**Anatomical site**					0.836
Anorectal	2 (18.2%)	2 (15.4%)	4 (36.4%)	8 (22.9%)	
Head & Neck	3 (27.3%)	4 (30.8%)	3 (27.3%)	10 (28.6%)	
Vulvovaginal	6 (54.5%)	7 (53.8%)	4 (36.4%)	17 (48.6%)	
**Breslow depth, mm**					0.228
Median (Q1,Q3)	3.0 (1.5, 8.6)	6.8 (4.5, 9.3)	10.5 (8.2, 12.8)	5.5 (2.9, 9.0)	

Unknown	2	5	9	16	
**Breslow depth, mm**					0.241
Unknown	2	5	9	16	
<5mm	5 (55.6%)	2 (25.0%)	0 (0.0%)	7 (36.8%)	
≥5mm	4 (44.4%)	6 (75.0%)	2 (100.0%)	12 (63.2%)	
**BRAF mutations**					0.781
No	9 (100.0%)	11 (84.6%)	10 (90.9%)	30 (90.9%)	
Yes	0 (0.0%)	2 (15.4%)	1 (9.1%)	3 (9.1%)	
Unknown	2	0	0	2	
**Nodal involvement**					< 0.001
No	11 (100.0%)	3 (23.1%)	1 (9.1%)	15 (42.9%)	
Yes	0 (0.0%)	10 (76.9%)	10 (90.9%)	20 (57.1%)	
**Nodal status assessment technique**					
CT	0	1 (10.0%)	3 (30.0%)	4 (20.0%)	
MRI	0	2 (20.0%)	2 (20.0%)	4 (20.0%)	
PET-CT	0	4 (40.0%)	5 (50.0%)	9 (45.0%)	
SLNB	0	3 (30.0%)	0 (0.0%)	3 (15.0%)	
Unknown	11	3	1	15	
**Metastasis**					< 0.001
No	11 (100.0%)	13 (100.0%)	0 (0.0%)	24 (68.6%)	
Yes	0 (0.0%)	0 (0.0%)	11 (100.0%)	11 (31.4%)	
**Surgical resection**					0.006
No	0 (0.0%)	3 (23.1%)	7 (63.6%)	10 (28.6%)	
Yes	11 (100.0%)	10 (76.9%)	4 (36.4%)	25 (71.4%)	
**Radiotherapy**					0.771
No	9 (81.8%)	9 (69.2%)	9 (81.8%)	27 (77.1%)	
Yes	2 (18.2%)	4 (30.8%)	2 (18.2%)	8 (22.9%)	

**Systemic therapy**					< 0.001
No	10 (90.9%)	2 (15.4%)	0 (0.0%)	12 (34.3%)	
Yes	1 (9.1%)	11 (84.6%)	11 (100.0%)	23 (65.7%)	
**Number of systemic treatment lines**					< 0.001
Median (Q1,Q3)	0.0 (0.0, 0.0)	2.0 (1.0, 3.0)	1.0 (1.0, 2.0)	1.0 (0.0, 2.0)	
**Follow-up/survival time, months**					0.063
Median (Q1,Q3)	35.0 (15.0, 58.0)	24.0 (9.0, 35.0)	10.0 (6.5, 18.0)	21.0 (8.0, 35.0)	
**Death**					0.033
No	7 (63.6%)	2 (15.4%)	2 (18.2%)	11 (31.4%)	
Yes	4 (36.4%)	11 (84.6%)	9 (81.8%)	24 (68.6%)	

Figure S1: Kaplan–Meier Curves for overall survival by anatomical site vagina vs vulva.

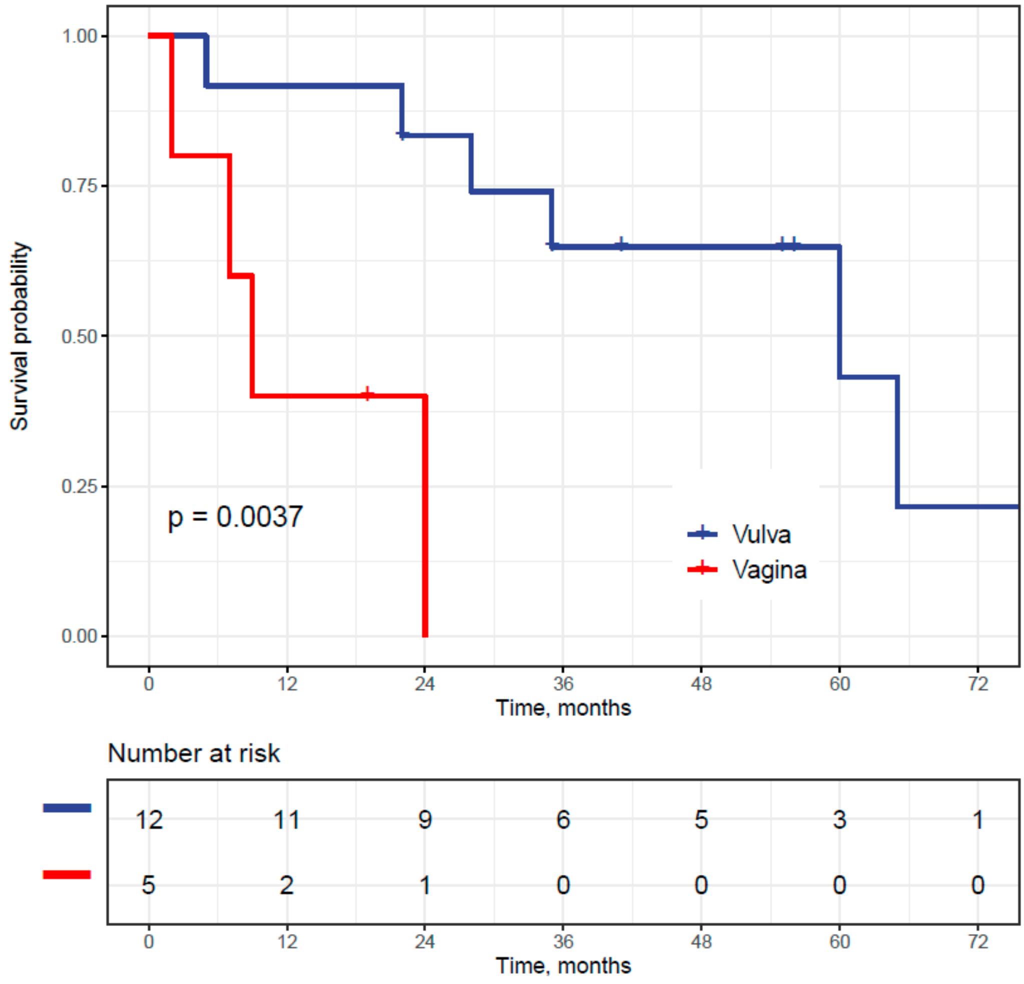

Supplement: Supplementary file 1 [file cancers-16-00227-s001.zip › cancers-2757297-supplementary.pdf]
